# Motor-system dynamics during naturalistic reading of action narratives in first and second language

**DOI:** 10.1016/j.neuroimage.2020.116820

**Published:** 2020-04-08

**Authors:** Agustina Birba, David Beltrán, Miguel Martorell Caro, Piergiorgio Trevisan, Boris Kogan, Lucas Sedeño, Agustín Ibáñez, Adolfo M. García

**Affiliations:** a Universidad de San Andrés, Buenos Aires, Argentina; b National Scientific and Technical Research Council (CONICET), Buenos Aires, C1425FQB, Argentina; c Instituto Universitario de Neurociencia (IUNE), Universidad de La Laguna, Tenerife, 3820, Spain; d University of Trieste, Italy; e Institute of Basic and Applied Psychology and Technology (IPSIBAT), National University of Mar del Plata, Buenos Aires, Argentina; f National Agency of Scientific and Technological Promotion (ANPCyT), Buenos Aires, Argentina; g Centre of Excellence in Cognition and Its Disorders, Australian Research Council (ARC), Sydney, NSW, 2109, Australia; h Center for Social and Cognitive Neuroscience (CSCN), School of Psychology, Universidad Adolfo Ibáñez, Santiago de Chile, 7550344, Chile; i Universidad Autónoma del Caribe, Barranquilla, 08002, Colombia; j Faculty of Education, National University of Cuyo, Mendoza, M5502JMA, Argentina; k Departamento de Lingüística y Literatura, Facultad de Humanidades, Universidad de Santiago de Chile, Santiago, Chile

**Keywords:** Embodied cognition, Action semantics, Naturalistic text reading, Bilingualism, EEG functional Connectivity

## Abstract

Do embodied semantic systems play different roles depending on when and how well a given language was learned? Emergent evidence suggests that this is the case for isolated, decontextualized stimuli, but no study has addressed the issue considering naturalistic narratives. Seeking to bridge this gap, we assessed motor-system dynamics in 26 Spanish-English bilinguals as they engaged in free, unconstrained reading of naturalistic action texts (ATs, highlighting the characters’ movements) and neutral texts (NTs, featuring low motility) in their first and second language (L1, L2). To explore functional connectivity spread over each reading session, we recorded ongoing high-density electroencephalographic signals and subjected them to functional connectivity analysis via a spatial clustering approach. Results showed that, in L1, AT (relative to NT) reading involved increased connectivity between left and right central electrodes consistently implicated in action-related processes, as well as distinct source-level modulations in motor regions. In L2, despite null group-level effects, enhanced motor-related connectivity during AT reading correlated positively with L2 proficiency and negatively with age of L2 learning. Taken together, these findings suggest that action simulations during unconstrained narrative reading involve neural couplings between motor-sensitive mechanisms, in proportion to how consolidated a language is. More generally, such evidence addresses recent calls to test the ecological validity of motor-resonance effects while offering new insights on their relation with experiential variables.

## 1. Introduction

Do embodied semantic systems play different roles depending on when and how well a given language was learned? This key question within the language grounding framework has been informed by bilingual experiments targeting action words in first and second languages (L1s, L2s) (Ahlberg et al., 2017; De Grauwe et al., 2014; Dudschig et al., 2014; Kogan et al., 2020; Vukovic and Shtyrov, 2014). However, the field is undermined by low ecological validity (i.e., low representativeness), as it relies mainly on decontextualized word-level stimuli presented in sequences that do not reflect the conditions of reading in daily life (García et al., 2018; Trevisan et al., 2017). To bridge this gap, the present study examines electroencephalographic (EEG) connectivity signatures of motor-system modulation in bilinguals with varying proficiency levels and ages of L2 learning as they freely read action-laden and non-action-laden narratives in L1 and L2.

Abundant research shows that, in L1, action-related words increase motor-network activity (Aziz-Zadeh et al., 2006; García et al., 2019; Hauk et al., 2004), modulate neurophysiological markers of action-language coupling (Aravena et al., 2010; Ibáñez et al., 2013), and affect ongoing physical movements (Bergen et al., 2010; García and Ibáñez, 2016a; Marino et al., 2014). Though scanter, evidence from action-language experiments in L2 has revealed similar behavioral (Buccino et al., 2017) and neurofunctional (Bergen et al., 2010; De Grauwe et al., 2014; Ibáñez et al., 2010; Vukovic, 2013; Vukovic and Shtyrov, 2014; Xue et al., 2015) effects, although these are weaker (Vukovic and Shtyrov, 2014) or less widespread (De Grauwe et al., 2014) than in L1. In fact, reduced embodied reactivations for L2 than L1 have also been reported during processing of emotion-related language (Foroni, 2015; Hsu et al., 2015). Therefore, as recently proposed in an integrative review (Kogan et al., 2020), the engagement of sensorimotor brain systems may be attenuated in languages learned after middle childhood (L2s) as compared with those acquired since intra-uterine life (L1s).

Moreover, the magnitude of embodied effects is further sensitive to L2-specific variables, crucially including L2 proficiency –i.e., the current level of ability in L2 usage (Hulstijn, 2012) – and age of L2 learning –i.e., the approximate period signaling language appropriation (Paradis, 2009). In fact, as captured in psycholinguistic (Dijkstra and van Heuven, 2002; Dijkstra et al., 2018; Kroll et al., 2010) and neuroscientific (Green, 2004; Paradis, 2009; Ullman, 2001) models, these two factors can modulate linguistic functions in bilinguals. For instance, higher proficiency levels are associated with greater reliance on direct (as opposed to L1-mediated) word-concept mappings in L2 (Guasch et al., 2008; Sunderman and Kroll, 2006), less asymmetric processing when comparing L1 and L2 tasks (García, 2015a; García et al., 2014), and more convergent recruitment of neural resources in both languages (Abutalebi, 2008). Similarly, a lower age of L2 learning has been linked to more parallel co-activation of both languages (Canseco-Gonzalez et al., 2010), increased inhibition of the non-target language (Bylund et al., 2019), greater neural sensitivity to fine-grained semantic distinctions in L2 (Vilas et al., 2019), and less marked differences in brain activation between L2 and L1 (Liu and Cao, 2016). More particularly, neurocognitive embodied effects during L2 action-language processing have been shown to correlate with L2 proficiency (Bergen et al., 2010) or even to be present only in high- as opposed to low-proficiency subjects (Ibáñez et al., 2010; Vukovic, 2013). Briefly, these findings suggest that, much like other neurolinguistic mechanisms, embodied systems could be differentially recruited depending on how well (and, possibly, when) a language has been learned.

Now, except for a few works that have explored comprehension of naturalistic narratives in bilinguals within the simulation theory framework (Adams et al., 2018; Walker et al., 2017), this empirical corpus is marked by a major shortcoming: its virtually null ecological validity. Indeed, while the above studies involved randomized series of disconnected and (pseudo)randomized items (for a review, see Kogan et al., 2020), daily language processing is based on context-rich texts characterized by cohesion, coherence, and unfolding semantic relations (Halliday and Matthiessen, 2014). Moreover, by requiring subjects to either read isolated words presented in quick flashes and/or respond to them with arbitrary movements –see García and Ibáñez (2016b) for a review, and Afonso et al. (2019) for a critique–, all those experiments fail to capture the actual conditions of written language processing, which typically involves reading multi-sentential paragraphs presented all at once (Hasson et al., 2018). Therefore, the bulk of embodied research is mostly moot on how, and even whether, grounding mechanisms are critically engaged during *naturalistic L1 and L2 processing*.

Promisingly, a new framework has begun illuminating the issue through the use of realistic yet highly controlled narratives (Trevisan and García, 2019). This approach has shown that action comprehension in naturalistic stories is distinctively and primarily affected in patients with movement disorders (García et al., 2018) and selectively boosted via sustained bodily training (Trevisan et al., 2017). Moreover, measures of ongoing brain signatures in other discourse-level paradigms suggests that action-related regions are modulated as a function of noun manipulability (Desai et al., 2016). Taken together, these findings suggest that motor systems do play a critical role in grounding action-related meanings embedded in naturalistic narratives. A fertile space is thus opened to assess the neural bases of these ecological phenomena in L1 and L2, as well as their relation with the subjects’ L2 proficiency and age of L2 learning.

Here we address this challenge focusing on free reading of paragraphlong narratives. EEG affords a valuable framework to this end, as it allows replicating typical conditions of everyday reading (assuming a sitting position while facing multi-sentential texts all at once) while targeting effects over specific task-sensitive electrodes. In this sense, modulations over left and right central electrodes –particularly those around electrodes C3 and C4, often termed ‘motor electrodes’ (Ewen et al., 2016; van Ede et al., 2019)– represent robust indexes of action-related processes, including object grasping (Ewen et al., 2016), normal (Edelman et al., 2015; Neuper et al., 2005; Pfurtscheller et al., 2006) and abnormal (López-Larraz et al., 2015) motor imagery, and action-verb access (Melloni et al., 2015; Vukovic and Shtyrov, 2014). Moreover, although an EEG setting poses major challenges to typical word-by-word analyses over long written passages (due to the tendency to look back at previous chunks of discourse, the difficulties of ascribing differential signatures between texts to any specific fine-grained variable, and the impossibility of tracking global neural states cutting across the reading act) (Picton et al., 2000), these limitations can be overcome by (i) using texts that are carefully controlled over multiple relevant dimensions and (ii) analyzing neural activity spread over the whole of each reading session. In particular, the latter requisite can be met via functional connectivity metrics, such as the weighted symbolic mutual information (wSMI) method, capable of discriminating between cognitive macro-states over extended time periods (Imperatori et al., 2019).

Building on this rationale, we conducted two tasks (one in L1, and one in L2) to examine functional connectivity signatures of embodied processing during naturalistic text reading. Specifically, in each language, we assessed cognitive macro-states spread over unrestricted reading of two types of narrative: an action text (AT), focused on the characters’ bodily movements, and a neutral text (NT), featuring low action content. Based on previous findings (De Grauwe et al., 2014; Ibáñez et al., 2010; Vukovic and Shtyrov, 2014), we hypothesized that, compared to the NT, the AT in L1 would involve greater connectivity among (motor-related) left and right central electrodes as well as distinct source-space activity modulations in motor regions. Also, we anticipated that enhanced motor-related connectivity would be attenuated in L2, but still associated with the subjects’ L2 proficiency and age of L2 learning. Briefly, with this innovative approach, we aim to illuminate how linguistic experience shapes sensorimotor grounding in highly ecological conditions.

## 2. Methods

### 2.1. Participants

The study comprised 30 bilinguals from Argentina who acquired Spanish as L1 and learned English as L2, mainly through formal, classroom-based instruction. They all had normal or corrected-to-normal vision and no history of neurological or psychiatric disease. Four subjects were excluded because of technical errors during signal recording. Thus, the final group consisted of 26 bilinguals, a sample size that reaches a power of .97 (see Supplementary material, section S1). The group was composed of 21 women and 5 men, with a mean of 30.30 (*SD* = 7.94, range: 19–46) years of age, an average age of L2 learning of 7.28 (*SD* = 3.52; range: 1–18), and 16.80 (*SD* = 7.35, range: 4–30) years of L2 study. Self-report data obtained through a previously reported questionnaire (Vilas et al., 2019) indicated that, on a scale from 1 (complete inability to perform even basic linguistic tasks) to 7 (high capacity to routinely deploy those skills at ease), the sample had high levels of proficiency in both languages (L1: *M* = 6.61, *SD* = 0.50, range: 6–7; L2: *M* = 6.07, *SD* = 0.84, range:4–7).

All participants read and signed an informed consent form before beginning the study. The protocol was carried out in accordance with the Declaration of Helsinki and was approved by the Ethical Research Committee of the Institute of Cognitive Neurology (Argentina), a host institution of the Institute of Cognitive and Translational Neuroscience.

### 2.2. Materials

The materials consisted of four simple short stories. All texts were built following a systematic protocol (Trevisan and García, 2019) and they were reported in previous studies (García et al., 2018; Trevisan et al., 2017). Two of them were created in Spanish (L1) and the other two were created in English (L2). Each pair was composed of one AT, which systematically focused on the characters’ bodily movements; and one NT, typified by low action content. Descriptive and statistical details about each text pair is offered in the next subsections.

#### 2.2.1. L1 task: Spanish stories

The two L1 stories, reproduced in the Supplementary material (section S2), were taken from García et al. (2018). The L1-AT narrates an afternoon in the life of Juancito, focusing on his bodily actions while he plays with his parents in a park. His activities include running on the grass, playing soccer, and interacting with both his parents and various objects. Also highlighted are the bodily actions of other characters, such as a clown who dances and children who applaud him. For example, one of the sentences reads “*Juancito corrió velozmente hacia el lugar donde el payaso saltaba y bailaba*” (“Juancito ran quickly to the place where the clown was jumping and dancing”). Also, the text includes rich details about the park, the objects in it, and how bodily actions were performed. A key aspect of this text is that 24 out of 32 of its verbs denote explicit motor actions. The other eight (non-action) verbs denote mental, relational, or emotional states (e.g., the boy falling asleep) as well as impersonal happenings (e.g., the sun coming up).

The L1-NT describes the feelings, thoughts, and perceptions of Alberto, a young man who is relaxing at a bar in a disco. Emphasis is placed on Alberto’s affective, mental, and sensory processes as he talks to his friend, Mario, and his girlfriend, Elsa. In particular, several sentences focus on his emotional responses to surrounding events [e.g., “*Alberto escuchó su canción favorita y se entusiasmó mucho*” (“Alberto heard his favorite song and was very excited”)]. Besides, the text offers abundant circumstantial information about places, objects, and temporal features of Alberto’s inner states. Crucially, 31 out of 32 verbs in the story denote non-motor processes. The remaining verb denoted bipedal movement (namely, crossing the street).

Importantly, the two narratives were comparable across multiple syntactic, lexical, semantic, pragmatic, and text-level variables, as in García et al. (2018). See Table 1 for statistical details.

#### 2.2.2. L2 task: English stories

The L2 stories, presented in the Supplementary material (section S3), were extracted from (Trevisan et al., 2017). The L2-AT narrates the day when Donald lost his moneybag, focusing on his bodily actions as he looks for it. His activities include running to his friend’s house and interacting with both his friend and various objects. The story also describes the bodily actions of other characters, such as a receptionist who types a newspaper ad. For example, one of the sentences reads “*He gave him the money and added some more coins*”. Also, the text specifies aspects of the locations and objects involved in the story, in addition to details about how physical actions were executed. Importantly, 25 out of 32 verbs in this AT refer to motor actions. The other seven verbs point to events that do not necessarily imply bodily movements, such as thinking or losing an object.

The L2-NT narrates the day when Steve discovered the taste of chocolate, focusing on his mental, sensory, and affective processes. The narration mostly revolves around his emotional responses to ongoing events (e.g., “*How much he loved them!*”). In addition, the text offers abundant circumstantial information depicting the village where Steve lives, the objects involved, and temporal features of his inner states. Importantly, the majority of verbs in this story (23 out of 32) denote non-motor processes. The remaining nine verbs allude to events that could be construed as requiring movement, such as going, teaching, and starting a journey. Furthermore, the number of action verbs in the L2-AT and non-action verbs in the L2-NT did not differ significantly from their counterparts in L1 [L1-AT action verbs = 24, L2-AT action verbs = 25, *X^2^*_(1)_*=* 0.02, *p* = .88; L1-NT non-action verbs = 31, L2-NT non-action verbs = 23, *X^2^*_(1)_ = 1.18, *p* = .27].

Crucially, the two L2 narratives were comparable across multiple syntactic, lexical, semantic, pragmatic, and text-level variables, as in Trevisan et al. (2017). See Table 2 for statistical details.

### 2.3. Procedure

**Fig. 1.**
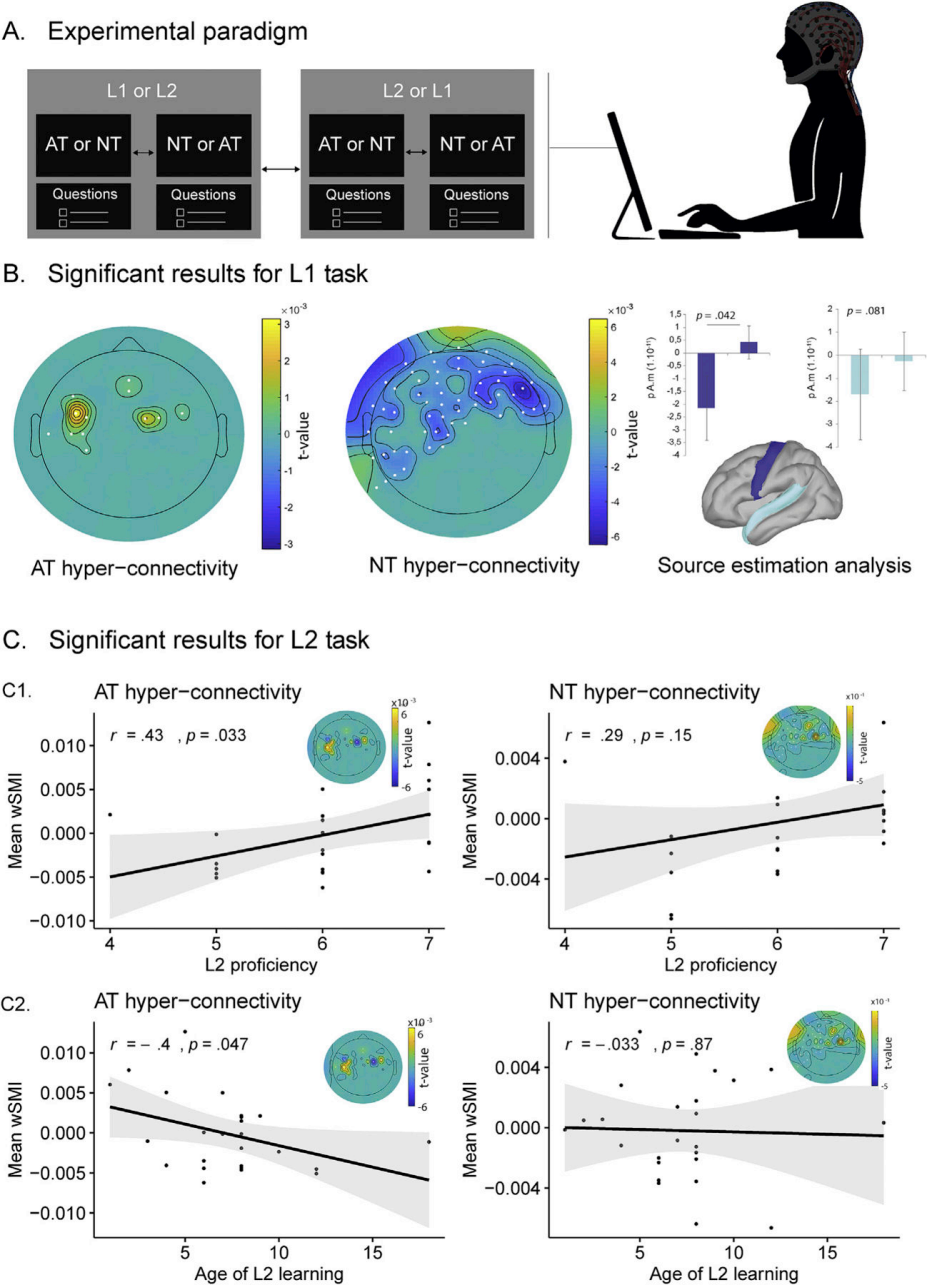
Experimental setup and significant results. **A.** Experimental paradigm. Participants read an AT and an NT in their L1 and their L2, each text being followed by three comprehension questions to force attentive reading. The order of the tasks (L1, L2) and of the texts within them (AT, NT) was counterbalanced across participants. **B.** Significant results for L1 task. The left and middle insets show the topographic wSMI patterns of the subtracted connectivity between AT and NT (0.5–11 Hz). Paired comparisons were performed between the AT and the NT (cluster-based non-parametric permutation test, *p* < .05). The panel shows enhanced connectivity patterns for the AT relative to the NT (left inset), and for the NT relative to the AT (middle inset). The right inset shows significant brain activation differences between the AT and the NT in a motor ROI (blue), together with non-significant differences for the same contrast in a temporal (non-motor) ROI (light blue). **C.** Significant results for L2 task. **C1.** Pearson’s correlation between L2 proficiency and enhanced L2-AT connectivity based on a data-driven action-grounding ROI (left inset), as well as between L2 proficiency and enhanced L2-NT connectivity based on a data-driven action-grounding ROI (right inset). **C2.** Pearson’s correlation between age of L2 learning and enhanced L2-AT connectivity based on a data-driven action-grounding ROI (left panel), as well as between age of L2 learning and enhanced L2-NT connectivity based on a data-driven action-grounding ROI (right panel). The graphs insets display the topographic wSMI of the subtracted connectivity between AT and NT (0.5–11 Hz) for each data-driven ROI, masked with significant results from the cluster-based analysis of L1 task. The color-bars of the topographs show the permutation test statistic for the difference between conditions, with yellow indicating higher connectivity during AT processing and violet denoting higher connectivity during NT processing. White dots represent the cluster’s significant electrodes. AT: action text; NT: neutral text; L1: first language; L2: second language.

First, participants completed the self-report questionnaire described in section 2.1. Then, they sat comfortably facing a computer screen in a dimly illuminated EEG room. They were told that they would be shown written texts on the screen, some in Spanish and some in English, and that they should simply read them silently, at their own pace and only once, without moving their heads, arms or bodies. They were further informed that, after reading each text, they would have to answer three comprehension questions (this was done to force attention and guarantee deep semantic processing throughout the task). The study began with a brief practice block, which consisted of one narrative with the same length and structure as the ones in the experiments, followed by three sample questions. After the practice block, participants read the AT and NT of one language and then the other two. Each text was presented all at once, in a single justified paragraph typed in white font (Calibri, size 22) against a black background. Participants were instructed to press a key to launch each text and then to remove it once they had finished reading it. The order of the tasks (L1, L2) and the sequencing of the AT and the NT within them, were systematically counterbalanced across participants –with the strategic constraint that two texts from the same category (e.g., the Spanish AT and the English AT) were never presented successively. Immediately after each narrative, the volunteer was presented with three true or false questions and asked to choose the correct answer by pressing predefined keys. Overall, the experiment lasted roughly 10 min. The structure of the experimental session is diagrammed in Fig. 1A.

### 2.4. EEG methods

#### 2.4.1. Preprocessing

During the reading of each text, EEG signals were acquired online through a Biosemi Active-two 128-channel system with pre-amplified sensors and a DC coupling amplifier, at a sampling rate of 1024 Hz. Analog filters were set at 0.03 and 100 Hz. A digital bandpass filter between 0.5 and 30 Hz was applied offline to remove unwanted frequency components. The reference was set to link mastoids for recordings and re-referenced offline to the average of all electrodes. Eye movements and blink artifacts were removed via independent component analysis and artifacts were rejected through visual inspection by an expert, following the exact same approach used by our team in previous EEG studies assessing linguistic and non-linguistic processes in diverse populations (Aravena et al., 2010; Dottori et al., 2017, 2020; García--Cordero et al., 2016; Ibáñez et al., 2010, 2013; Melloni et al., 2015; Vilas et al., 2019; Yoris et al., 2017). Bad channels were replaced with statistically weighted spherical interpolation (based on all sensors) and then the variance of the signal across trials was calculated to guarantee the stability of the averaged waveform (Courellis et al., 2016). Events were inserted every 1 s from the beginning until the end of the reading of each text, resulting in four type of events: action text in L1 (L1-AT), neutral text in L1 (L1-NT), action text in L2 (L2-AT), and neutral text in L2 (L2-NT). The number of events of each text depended on the time the participant took to read the text. Importantly, averaged reading latencies did not differ significantly [*F*(3,75) = 1.48, *p* = .23] among the four texts (L1-AT: *M* = 58.60 s, *SD* = 14.54 s; L1-NT: *M* = 66.23 s, *SD* = 18.00 s; L2-AT: *M* = 64.14 s, *SD* = 21.40 s; L2-NT: *M* = 60.87 s, *SD* = 23.22 s). Likewise, the number of data segments was similar [*F*(3,75) = 1.45, *p* = .25] across the four texts (L1-AT: *M* = 62.65, *SD* = 3.93; L1-NT: *M* = 69.57, *SD* = 3.93; L2-AT: *M* = 68.46, *SD* = 3.93; L2-NT: *M* = 63.96, *SD* = 3.93). As done in previous EEG studies employing the wSMI method to Complex/complex-compound sentences examine temporally variable cognitive states (Imperatori et al., 2019), we selected 1-s segments from continuous data. These processing steps were implemented using custom MATLAB scripts based on EEGLAB toolbox (Delorme and Makeig, 2004) and custom-made scripts for further processing.

#### 2.4.2. Weighted symbolic mutual information (wSMI) methods

The wSMI metric is a functional connectivity method that captures patterns of non-linear information sharing. Of note, this method is sensitive capture modulations that escape the possibilities of strictly linear methods, such as correlations or the coherence metric. Moreover, it has proven sensitive to track different higher-order cognitive operations (García-Cordero et al., 2017; Hesse et al., 2015), including general (Hesse et al., 2019) and embodied (Melloni et al., 2015) semantic processes, as well as cumulative differences between contrastive cognitive states over varying periods of time (Imperatori et al., 2019). In line with standard procedures (King et al., 2013), signals were transformed into symbols. By defining values of *k* (the number of samples that represent a symbol, set to 3) and τ (the temporal separation between them, set to 32 ms), we sensitized wSMI to a frequency range of 0.5–11 Hz, which is apt to capture modulations related to motoric (Pfurtscheller and Neuper, 1997) and action-semantic (Vukovic and Shtyrov, 2014) processes, while also discriminating among low- and high-proficiency bilinguals during naturalistic discourse processing in L1 and L2 (Reiterer et al., 2005). The joint probability between the signals was then calculated for each pair of channels, for each data segment, and wSMI was estimated using a joint probability matrix multiplied by binary weights. These weights were set to zero for pairs of (a) identical symbols and (b) opposed symbols that could be elicited by a unique common source.

#### 2.4.3. Exploratory source estimation analysis

Brainstorm’s Matlab toolbox was used to estimate source activations from scalp EEG activity (Tadel et al., 2011). The 128 sensor positions of Biosemi’s cap were aligned to an anatomy template created from the standard MNI-152 template (ICBM-152, without white-matter envelope). A forward lead field (or gain) model, composed of 15000 vertices distributed along the cortical surface, was computed using the Open-MEEG boundary element model (Gramfort et al., 2010). For each participant and text condition, this common lead field model was combined with observed EEG activity to solve the inverse problem (i.e., estimation of source activity) using the standardized Low Resolution Electromagnetic Tomography (sLORETA) method (Pascual-Marqui, 2002). Resulting activity in the source space was grouped according to the 62 brain regions parceled by the Mindboggle Atlas (Klein and Hirsch, 2005). In particular, following previous action-language research (García et al., 2019), we selected four relevant scouts namely: a left motor (precentral) scout, a right motor (precentral) scout, a left superior temporal scout, and a right superior temporal scout. The activity of each scout was averaged across time. Specifically, to test the prediction that the AT would elicit greater motor activation than the NT, we conducted one-tailed *t*-tests considering the activity underlying each text in each scout.

### 2.5. Data analysis

All analyses were performed for each task independently, comparing the L1-AT vs. the L1-NT, on the one hand, and the L2-AT vs. the L2-NT, on the other. To analyze the connectivity matrix of each participant in each task, we performed a nonparametric cluster-based permutation test for dependent samples (Maris and Oostenveld, 2007), an approach that has proven sensitive to semantic effects (Moreno et al., 2015), even in bilinguals (Vilas et al., 2019). In both the L1 task and the L2 task, whole-brain connectivity links were also evaluated and compared between AT and NT data via non-parametric permutation tests (with 1000 iterations). Electrode pairs were considered part of the same cluster if their connectivity reached *p <* .05. These clusters were considered significant with a minimum of ten electrodes and at a *p* < .025 (assuming an alpha level of 0.05) relative to the calculated sample, as in previous works (Maris and Oostenveld, 2007; Vilas et al., 2019). The significant clusters were used to mask the raw matrix. Then, for each language, the NT data was subtracted from the AT data and plotted in a topography plot, with positive values of the cluster representing greater connectivity for the AT and negative values representing greater connectivity for the NT.

Moreover, to evaluate whether the recruitment of embodied mechanisms was related to the degree of entrenchment of the L2, we performed Pearson’s correlations between the connectivity of motor regions and the participants’ (a) L2 proficiency and (b) age of L2 learning. For maximal stringency in our analysis, correlations were replicated over three estimations of motor connectivity. First, we derived a data-driven ROI based on the results of the cluster analysis. Specifically, given that no significant clusters were observed for the L2 task (see section 3.2.1), we established a motor ROI comprising the electrodes that showed enhanced connectivity during L1-AT processing [B30, B32, C2, C20, C21, D5, D11, D12, D20, D22, D28] (Supplementary data, section S4, Figure S1A) and used it to mask the connectivity matrix of the L2 conditions. This analysis was complemented with a control ROI comprised of the electrodes yielding enhanced connectivity during L1-NT processing [B27, B28, B29, B31, C1, C4, C5, C6, C7, C8, C9, C10, C11, C12, C13, C14, C15, C16, C17, C18, C22, C23, C24, C25, C26, C27, C28, C29, C30, C31, C32, D1, D2, D3, D4, D6, D7, D8, D9, D10, D13, D14, D16, D17, D18, D21, D23, D24, D25, D26, D27, D29, D30, D31, D32] (Supplementary data, section S4, Figure S1A). Second, we replicated the correlation analysis using two hypothesis-driven ROIs, namely: an embodied ROI derived from a previous study on action semantics [D12, D19, D28] (Vukovic and Shtyrov, 2014) (Supplementary data, section S4, Figure S1B movement-sensitive ROI taken from a motor-tapping experiment [ D11, D12, D13, D18, D19, D20] (Yoris et al., 2017) (Supplementary data, section S4, Figure S1C). Then, for each subject, we averaged the connectivity of all the electrodes in the ROI upon subtraction of the AT from the NT. On the other hand, for the two hypothesis-driven ROIs, we directly averaged the connectivity of all electrodes of the ROI upon subtraction of the AT from the NT, without masking the connectivity matrix. Moreover, to ensure that the predicted correlations were specific to L2 embodiment, we analyzed whether L2 proficiency and age of L2 learning were related to enhanced connectivity during (i) L2-NT processing and (ii) L1-AT processing. The cluster analysis was performed with Matlab software and the Pearson’s correlations were run on R 3.5.2 software.

## 3. Results

### 3.1. L1 task

#### 3.1.1. Cluster analysis in L1

Comparisons of brain activity between the AT and NT in L1 revealed significantly different clusters (*p* = .005, cluster-corrected; Fig. 1B, left and middle insets). Specifically, the AT presented increased connectivity between left and right motor-related electrodes (Fig. 1B, left inset), while the NT exhibited higher connectivity over the rest of the cluster’s significant electrodes, covering left and right frontal and temporal locations (Fig. 1B, middle inset).

#### 3.1.2. Exploratory source estimation results

The contrast between AT and NT activity in L1 revealed significant differences over the left motor scout [*t*(43.411) = 1.77, *p* = .04], with no significant effects over the left temporal scout [*t*(40.31) = 1.42, *p* =.08] –Fig. 1B, right inset. Neither did we find any significant differences in the right motor scout [*t*(45.959) = 1.68, *p* = .95] or the right temporal scout [*t*(39.594) = −0.59742, *p* = .28].

### 3.2. L2 task

#### 3.2.1. Cluster analysis in L2

The direct contrast between AT and NT revealed no significant functional connectivity differences in L2 (*p* > .025, cluster-corrected).

#### 3.2.2. Correlations between enhanced AT connectivity in L2 and measures of L2 entrenchment

Despite the lack of connectivity differences between the L2 texts when averaging the whole sample, we examined whether enhanced connectivity among motor-related electrodes during AT processing was related to measures of L2 entrenchment (namely, L2 proficiency and age of L2 learning). Results from our data-driven approach revealed a positive correlation between L2 proficiency and enhanced connectivity of the AT-based ROI in L2 (AT vs. NT) (*r* = 0.43, *p* = .03, Fig. 1C1, left inset) but not with enhanced connectivity of the NT-based ROI (*r* = 0.29, *p* =.15, Fig. 1C1, right inset). These results were specific to L2, since the correlations between L2 proficiency and the connectivity of the AT- and NT-based ROIs in L1 (AT vs. NT) processing did not reveal any significant results (see Supplementary material, section S5). These results were replicated by the correlations based on hypothesis-driven ROIs, as L2 proficiency positively correlated with the connectivity of the embodied ROI (*r* = 0.43, *p* =.03) and of the movement-sensitive ROI (*r* = 0.41, *p* = .03) during L2 (AT vs. NT) processing. These results were also specific to L2, since the correlations between L2 proficiency and the connectivity of the AT- and NT-based ROI during L1 (AT vs. NT) proficiency did not reveal any significant results (Supplementary material, section S5).

Additionally, we inspected the relation between age of L2 learning and enhanced connectivity for AT and NT in L2. We found a negative correlation with the data-driven AT ROI (*r* = 0.40, *p* = .047, Fig. 1C2 left panel) but not with the connectivity of the data-driven NT ROI (*r* = −0.03, *p* =.87, Fig. 1C2, right panel). As in the analysis of L2 proficiency, these results were specific to L2, since no significant correlations were observed with the connectivity of either ROI during L1 (AT vs. NT) processing (Supplementary material, section S6). However, the significant correlation observed for the data-driven AT ROI was not replicated in the analyses based on the two hypothesis-driven ROIs, namely: the embodied ROI (*r= −*0.25, *p* =.21) and the movement-sensitive ROI (*r = −*0.23, *p* =.26). Furthermore, we reran these analyses after removing one subject whose age of L2 learning (namely, 18) was found to be an outlier at 2 *SD*s from the sample’s mean. The correlation between connectivity in the AT ROI and age of L2 learning remained significant, whereas all control correlations remained non-significant (for details, see Supplementary material, section S6, Figure S2).

## 4. Discussion

This study examined whether the recruitment of embodied semantic systems during naturalistic text reading is driven by when and how well a language was learned. During L1 processing, AT reading involved increased connectivity between left and right (motor-related) central electrodes, together with differential activation of motor regions. Moreover, although no such pattern was observed for L2 when collapsing all participants, enhanced motor-related connectivity during L2-AT processing correlated positively with L2 proficiency and negatively with age of L2 learning. Crucially, all these patterns were specific to AT (as opposed to NT) reading. Therefore, the role of embodied semantic systems during naturalistic discourse processing seems sensitive to ontogenetic and language proficiency factors.

The principal finding in the L1 task is that reading of the L1-AT involved increased connectivity between left and right central electrodes. Crucially, the electrodes in this cluster have been consistently linked to signatures of action-related processes, like event-related desynchronization of the beta band during object grasping (Ewen et al., 2016), changes of oscillatory activity in motor imagery tasks (Edelman et al., 2015; Neuper et al., 2005; Pfurtscheller et al., 2006), and disruptions of such patterns in sub-acute tetraplegic patients (López-Larraz et al., 2015). More particularly, in previous studies, similar sets of electrodes have shown modulations of mu rhythms (Vukovic and Shtyrov, 2014) and the motor potential (Melloni et al., 2015) during action-verb access. Considering the specific manipulation between the AT and the NT, this pattern suggests that action comprehension may distinctively recruit motor mechanisms, as previously indicated by neuroimaging (Aziz-Zadeh et al., 2006; García et al., 2019; Hauk et al., 2004), electrophysiological (Aravena et al., 2010; Ibáñez et al., 2013), and behavioral (Bergen et al., 2010; García and Ibáñez, 2016a; Marino et al., 2014) studies examining action-verb processing via isolated words and sentences, sometimes even in combination with actual physical movements –as seen, for instance, in studies showing faster knob-turning movements upon reading directionally compatible action sentences (Zwaan and Taylor, 2006). In line with canonical perspectives in the embodied semantics framework, this suggests that language comprehension is mediated by tacit reenactments of the sensorimotor experiences evoked by the verbal material at hand (Gallese and Cuccio, 2018; Gallese and Sinigaglia, 2011; Pulvermüller, 2013a,b, 2018).

Importantly, significant information exchange across such motor-related electrodes was specific to AT processing, as the L1-NT was typified by increased connectivity over left and right frontal and temporal electrodes (rather than those more typically associated with motoric processes). This selective pattern of motor grounding for the AT mirrors previous results based on the same naturalistic texts in L1 users. Indeed, motor dysfunction (García et al., 2018) and sustained bodily training (Trevisan et al., 2017) have been shown to respectively impair and boost comprehension of actions in L1-ATs without comparable effects in fully matched NTs. Accordingly, the pattern of enhanced connectivity observed here for the AT relative to the NT seems to specifically reflect motor grounding effects rather than unspecific markers of text reading at large.

Also, our exploratory source estimation analysis revealed differential modulations between the AT and the NT in left motor regions, with no such effects in right motor and bilateral temporal regions. This result aligns with abundant neuroimaging evidence showing predominant left-sided motor-region activations for action-verb processing (Boulenger et al., 2012; Mollo et al., 2016; Shtyrov et al., 2014; Willems et al., 2010), often accompanied by null (Berlingeri et al., 2008; Liljestrom et al., 2008; Raposo et al., 2009; Ruschemeyer et al., 2007) or non-primary (García et al., 2019) participation of temporal regions –but see Bedny et al. (2008) and Tyler et al. (2003). Taken together, these results further attest to the embodied nature of the connectivity results described above.

Of note, these findings constitute novel evidence of distinct non-linear *information sharing* between motor mechanisms during action-language processing. In this sense, the use of functional connectivity metrics for embodied language research (Abrevaya et al., 2017; García et al., 2016; Melloni et al., 2015) allows complementing classical single evoked-response approaches with much-needed insights on relevant cross-regional patterns (Mišić and Sporns, 2016). More particularly, it would seem that diverse motor mechanisms operate in dynamic coordination rather than in isolation to ground modality-specific meanings during language processing. In addition, and more crucially, they indicate that such coupling of embodied systems plays a critical role during *unconstrained reading of naturalistic narratives*. Importantly, this finding adds unprecedented neuroscientific support to the claim that motor-grounding mechanisms may be robust enough to emerge even in ecological scenarios (García et al., 2018; Trevisan et al., 2017).

Additional insights come from the L2 task. First, no significantly different clusters were observed between the L2-AT and the L2-NT when collapsing all participants. Though no study has evaluated wSMI connectivity in the same frequency range that we have tested, our result aligns with previous reports showing that other neural markers of embodiment, beyond functional connectivity, are attenuated in L2 relative to L1. For example, significant mu-rhythm modulations (a cortical marker of motor activity) during action-verb processing have been shown to be present in L1 but absent in L2 (Vukovic and Shtyrov, 2014), and the same is true of the recruitment of premotor areas as a complement to primary motor regions (De Grauwe et al., 2014). In line with previous studies (Chee, 2009; García, 2015b; Klein et al., 2006; Lucas et al., 2004; Ojemann and Whitaker, 1978; Paradis, 1989, 2009; Ullman, 2001), this shows that putative mechanisms underlying L1 processing are not necessarily shared by comparable L2 tasks across bilinguals at large.

However, and contrary to previous claims (Pavlenko, 2012), it does not follow that L2 processing is “disembodied” across the bilingual population. Quite on the contrary, our results suggest that the recruitment of embodied mechanisms during L2 reading depends on how entrenched the language is (Kogan et al., 2020; Monaco et al., 2019). Indeed, enhanced motor-related connectivity during L2-AT processing was positively correlated with L2 proficiency –a pattern that was replicated over alternative ROIs derived from motor (Yoris et al., 2017) and action-semantics (Vukovic and Shtyrov, 2014) tasks, and absent in control correlations with L2-NT connectivity. Compatibly, previous research has shown that greater N400 modulations for L2 action-related expressions accompanied by incongruent gestures emerged only in bilinguals with high (as opposed to low) L2 proficiency (Ibáñez et al., 2010). Moreover, effector-specific interference during action-word processing was observed in high-(but not in low-) proficiency bilinguals during image-verb matching (Bergen et al., 2010) and translation equivalent recognition (Vukovic, 2013). As shown for other comparisons between a bilingual’s two languages (Liu and Cao, 2016) or between low- and-high proficiency L2 users (Oh et al., 2019), this suggests that the recruitment of embodied systems during ecological L2 processing is related to how entrenched that language is.

In the same vein, enhanced motor-related connectivity during L2-AT processing was negatively correlated with age of L2 learning –a pattern that was also highly specific, as no significant results emerged from the control correlations. This pattern fits well with an extensive literature showing that age of L2 learning modulates multiple aspects of language processing, including semantic effects (Sabourin et al., 2014; Vilas et al., 2019). Nevertheless, as observed in other verbal (Consonni et al., 2013; De Carli et al., 2015; Green and acquisition, 2003) and non-verbal (De Carli et al., 2015) tasks, age of L2 learning may not be as robust as L2 proficiency in modulating neurocognitive effects. In this sense, note that the significant correlations based on the data-driven ROI were replicated with hypothesis-driven ROIs in the case of L2 proficiency, but not in the case of age of L2 learning. Tentatively, this could suggest that sensorimotor grounding during naturalistic L2 processing is more crucially driven by how well than by how early a language was learned. However, this conjecture should be directly tested in future studies.

Be that as it may, it is worth noting that connectivity results in both tasks were captured within the 0.5–11 Hz range. This range subsumes frequency bands implicated in general semantics, embodied semantics (including action language and action imagery), and motor planning and execution, as revealed through measures of oscillatory activity or functional connectivity (Babiloni et al., 2016, 2017; De Lange et al., 2008; Ewald et al., 2012; Hanouneh et al., 2018; Kikuchi et al., 2011; Moreno et al., 2013), including wSMI signatures of action-language coupling (Melloni et al., 2015). Moreover, analyses of functional connectivity (Elmer and Kühnis, 2016; Reiterer et al., 2005) and oscillatory activity (Grabner et al., 2007; Vilas et al., 2019; Vukovic and Shtyrov, 2014) within this frequency range have indexed differential semantic effects in L1 and L2, and even distinct patterns of language embodiment in such languages (Vukovic and Shtyrov, 2014). Therefore, our findings suggest that the same frequency range indexing relevant effects in word-level or otherwise atomistic paradigms is also distinctively engaged in ecological reading settings.

From a broader theoretical perspective, our findings underscore the importance of factoring in individual experience as a key determinant of language grounding mechanisms. As shown elsewhere, specific language embodiment phenomena (including relevant connectivity patterns) are driven by individuals’ athletic skills (Beilock et al., 2008; Tomasino et al., 2012, 2013), their level of dexterity (Locatelli et al., 2012) or difficulty (Abrevaya et al., 2017; Birba et al., 2017; García et al., 2016) in performing motor actions, and their degree of bodily engagement during classroom-based L2 learning (Macedonia and Klimesch, 2014). Of note, the latter point has been shown even with naturalistic texts. Indeed, in the “Moved by Reading” paradigm (Adams et al., 2018; Walker et al., 2017), children engage in embodied simulations by first moving computer images through physical actions that reflect the meaning of sentences in a text, and later creating internal simulations of the text via imagery. Upon doing so, children exhibit significantly better comprehension than controls (Adams et al., 2018), as long as they are good at word decoding (Walker et al., 2017). In line with these antecedents, our research indicates that earliness of language exposure and attained proficiency also represent subject-level variables modulating embodied effects. Taken together, such evidence emphasizes the futility of theoretical positions that frame language processing as either entirely embodied or entirely disembodied (Pavlenko, 2012; Pulvermüller, 2013a,b). Instead, they support more nuanced conceptualizations according to which the role of grounding effects depends on each person’s linguistic and motoric experiences (Ahlberg et al., 2017; Gramann, 2013; Repetto et al., 2015).

Admittedly, in this sense, our assessment of individual language profiles (particularly including proficiency estimations) is partly undermined by the use of self-reports. Despite their widespread use in bilingualism research (Hulstijn, 2012), subjective measures of L2 proficiency are susceptible to social desirability and self-image biases. Notably, however, they can reliably predict language ability (Marian et al., 2007), mirror reaction-time results in L2 tasks (Langdon et al., 2005), and even replicate scores in multilingual naming tests (Gollan et al., 2012). However, as shown by Tomoschuk et al. (2019), self-ratings of L2 proficiency and objective performance may not always pattern together, particularly when participants prove heterogeneous in their language combinations, cultural profiles, and patterns of language dominance. Although our study partly circumvents these issues by presenting a sample made up exclusively of Spanish-English bilinguals from the same socio-geographical setting, this does not fully rule out the biases and imprecisions mentioned above. Also, even though the specific questionnaire we used has been successfully employed to both separate and match bilingual samples based on language-experience factors (Santilli et al., 2018; Vilas et al., 2019), it lacks items separately tapping on each macro-skill (speaking, listening, reading, writing). Fortunately, these shortcomings could be bridged in future replications by incorporating more detailed self-report tools, such as the Language History Questionnaire (Li et al., 2006; Li et al., 2014; Li et al., 2019), ideally in tandem with objective proficiency measures –for a review, see Hulstijn (2012).

Finally, above and beyond these reservations, the importance of detecting embodiment effects in an unconstrained text reading task cannot be overemphasized. So far, all neurophysiological embodied research on bilinguals has relied on isolated, randomly sequenced words or sentences (Bergen et al., 2010; Buccino et al., 2017; De Grauwe et al., 2014; Ibáñez et al., 2010; Vukovic, 2013; Vukovic and Shtyrov, 2014; Xue et al., 2015). Though variously informative, such findings cannot be a priori assumed to hold during comprehension of context-rich, coherent and cohesive texts, given that contextual information modulates action-word processing (García and Ibáñez, 2016b; Van Dam et al., 2010) and variously affects linguistic performance by favoring maintenance of relevant information (Ledoux et al., 2006). Here, the presence of embodied effects spread over the reading of entire narratives, rather than simply locked to individual items within them (e.g., Desai et al., 2016), suggests that language-induced action simulations are robust enough to cut across the manifold textual richness characterizing naturalistic discourse. More particularly, their emergence in unconstrained reading settings indicates that such phenomena are not confined to the artificial processing conditions of laboratory settings, attesting to the potential translational relevance of the embodied framework at large (García et al., 2018; Trevisan et al., 2017).

## 5. Limitations and avenues for further research

A number of limitations can be identified in the present study, paving the way for future investigation. First, despite reaching high statistical power and surpassing the *N*s of several relevant studies (De Grauwe et al., 2014; Trevisan et al., 2017; Vukovic and Shtyrov, 2014), our sample size was modest, inviting replications with larger groups. Second, though validated (Trevisan and García, 2019) and objectively sensitive to embodied effects (García et al., 2018; Trevisan et al., 2017), the texts used in both tasks were relatively short and easy. It would thus be interesting to conduct further research incorporating longer and more varied texts. Third, given the idiosyncratic differences between Spanish and English (and hence, between the texts employed for each language), we were not able to directly compare neural signatures of L1 and L2. In this sense, new applications of the protocol detailed in Trevisan and García (2019) should aim to construct text pairs that are comparable both within and between languages, thus allow for direct inter-linguistic comparisons. Fourth, though the L1-NT and L2-NT were statistically comparable in their number of non-action verbs, such figures were not identical. Granted, both NTs have proven to be impervious to motor-resonance effects in their corresponding languages, as shown by evidence of intact non-action verb comprehension in Spanish following motor-network damage (García et al., 2018) and null effects of ecological bodily training on English non-action verb comprehension (Trevisan et al., 2017). However, future implementations of the present text-construction protocol for bilingualism research should strive to further improve the comparability of this variable between NTs in each language. Fifth, despite its clear advantages, the connectivity metric we employed presents objective drawbacks. In particular, wSMI does not allow analyzing isolated frequency ranges and it may lead to partial information loss by favoring relative over absolute differences between data points (Lee et al., 2015), thus partly reducing sensitivity to certain connectivity patterns (Casali et al., 2013; Gourévitch and Eggermont, 2007). In this sense, future studies may seek to reproduce these results using a different connectivity method. Finally, despite responding to a strategic methodological decision, the unconstrained reading task generates a bulk of known shortcomings in EEG recordings, mainly due to ocular/motor artifacts reflecting uncontrolled fixation and regression patterns during reading (Picton et al., 2000). Future research should aim to replicate this study with simultaneous eye-tracking recordings, in order to better estimate the role of reading-specific patterns across subjects. In this sense, incorporation of automatic artifact rejection methods could also be a valuable strategy to pursue so as to reduce preprocessing time and minimize the room for human errors.

## 6. Conclusion

In conclusion, our results indicate that embodied semantic systems play a critical role during unconstrained reading of naturalistic narratives in L1, and that the recruitment of such systems in L2 is associated with how well and how early that language was learned. Taken together, these findings address recent calls to test the ecological validity of motor-resonance effects while offering new insights on their relation with experiential variables. More generally, further efforts along these lines could strengthen the empirical integration of embodied and situated frameworks in cognitive science.

## Supplementary Material

1

## Figures and Tables

**Table 1:** Linguistic features of the action and neutral texts from L1 task (Spanish).

	Action text	Neutral text	Statistic	*p*-value*
Characters^a^	944	978	χ^2^ = .60	.44
Words	208	204	χ^2^ =.04	.84
Nouns	48	44	χ^2^ =.17	.68
Adjectives	7	9	χ^2^ = .25	.62
Adverbs	6	8	χ^2^ =.29	.59
****Verbs****	****32****	****32****	χ^2^ = 0	****1****
****Action verbs****	****24****	****1****	χ^2^ = 21.16	****< .001****
****Non-action verbs****	****8****	****31****	χ^2^ = 13.56	****< .001****
Mean content word frequency^b^	1.63	1.79	*t* = 1.53	.13
Mean content word familiarity^b^	6.15	6.24	*t* =.74	.46
Mean content word imageability^c^	5.25	4.97	*t* =1.39	.17
Mean content word syllabic length^c^	2.52	2.49	*t* =.25	.80
Mean content word orthographic length^c^	6.16	6.26	*t* =.36	.72
Sentences	22	22	χ^2^ = 0	1
Minor sentences	3	3	χ^2^ = 0	1
Simple sentences	8	8	χ^2^ = 0	1
Compound sentences	4	3	χ^2^ = .14	.71
Complex/complex-compound sentences	7	8	χ^2^ =.07	.80
Coherence	4.05	3.86	*t* = .62	.54
Comprehensibility	4.24	4.10	*t* = 1.05	.30
Readability (Szigriszt-Pazos Index)^d^	79.92	77.3	χ^2^ = .04	.83
Readability (Inflezs scale rating)^e^	Fairly easy	Fairly easy	–	–

# *p*-values calculated with chi-squared test. Alpha level set at .05.

* *p*-values calculated with independent measures ANOVA. Alpha level set at .05.

a Character count performed without counting spaces.

b Psycholinguistic data extracted from the LEXESP database, through B-Pal (Davis and Perea, 2005).

c Frequency data extracted from B-Pal (Davis and Perea, 2005).

d Formula applied as described in (Szigriszt Pazos, 1993).

e Formula applied as described in (Barrio-Cantalejo et al., 2008).

**Table 2:** Linguistic features of the action and neutral texts from L2 task (English).

	Action text	Neutral text	Statistic	*p*-value
Characters^a^	696	743	χ^2^ = 1.53	.215^#^
Words	167	169	χ^2^ = 0.01	.913^#^
Nouns	33	25	χ^2^ = 1.10	.293^#^
Adjectives	6	14	χ^2^ = 3.20	.073^#^
Adverbs	6	16	χ^2^ = 4.54	.3^#^
****Verbs****	****32****	****32****	χ^2^ = 0	**.999**^#^
****Action verbs****	****25****	****9****	χ^2^ = 5.44	**.020**^#^
****Non-action verbs****	****7****	****23****	χ^2^ = 7	**.008**^#^
Mean content word frequency^b,c^	802.05	974.6	*t* = 0.71	.474*
Mean content word familiarity^b,d^	593.2	582.4	*t* = 1.63	.104*
Mean content word imageability^b,e^	442.8	394.9	*t* = 1.98	.07*
Mean content word syllabic length^b^	1.3	1.5	*t* = 1.60	.111*
Mean content word orthographic length^b^	4.8	5.1	*t* = 1.98	.324*
Sentences	17	17	χ^2^ = 0	.999^#^
Minor sentences	0	0	χ^2^ = 0	.999^#^
Compound sentences	3	3	χ^2^ =.0	.999^#^
Complex/complex-compound sentences	7	6	χ^2^ =.07	.80
Comprehensibility	3.9	3.6	*t* = 0.684	.502*
Coherence	3.7	3.6	*t* = 0.186	.855*
Readability (PSKF)^f^				
	4.4	4.55	4.22	–
Readability (SRI)^g^	3	2.8	3.5	–

# *p*-values calculated with chi-squared test. Alpha level set at .05.

* *p*-values calculated with independent measures ANOVA. Alpha level set at .05.

a Character count performed without counting spaces.

b Psycholinguistic data extracted from N-Watch (Davis, 2005), based on lemma counts.

c Frequency data extracted from the CELEX written database, through N-Watch (Davis, 2005).

d Familiarity data extracted from the MRC database, through N-Watch (Davis, 2005).

e Imageability data extracted from the Bristol/MRC database, through N-Watch (Davis, 2005).

f Calculated through the Powers-Sumner-Kearl Formula (PSKF).

g Calculated through the Spache Readability Index (SRI) revised.
