# Supplementary material for: Motor-system dynamics during naturalistic reading of action narratives in first and second language
Source: Neuroimage. Author manuscript; Available in PMC 2020 Aug 7. (PMC7412856; doi:10.1016/j.neuroimage.2020.116820)
Supplement: 1 [file NIHMS1611110-supplement-1.docx]

**Supplementary materials**

**S1. Sample size estimation**

To determine the sample size required for our experiment, we ran an estimation analysis in R software with the pwr library. Given our statistical design, based on paired *t*-tests, we considered three parameters. First, we established alpha level of *p* = .05. Second, we considered an effect size of 0.80 (based on Cohen’s *d*). Finally, we established a power of 0.8. This analysis showed that a sample size of 14 is enough to reach the estimated effects. The final sample size of 26 reaches a power of .97.

**S2. Texts used in the L1 task**

**Action text:** Sábado por la tarde. ¡El momento favorito de Juancito en toda la semana! Tomó a sus padres de la mano y juntos corrieron hasta la plazoleta. Al lado de las hamacas, un grupo de niños aplaudía las ocurrencias de un colorido payaso. Juancito corrió velozmente hacia el lugar donde el payaso saltaba y bailaba sin cesar. Al terminar el espectáculo, el payaso escribió su nombre en el pavimento. ¡Qué sorpresa! ¡También se llamaba Juan! Luego, entre toda la muchedumbre, Juancito caminó hacia el banco donde se sentaron sus padres. Abrazó a su padre con mucha fuerza y le jaló la camisa para que se levantara. ¡Era hora de jugar al fútbol! Juancito tomó la pelota y la puso en el césped. Su padre se movía de izquierda a derecha, en posición de arquero. Juancito pateó y… ¡gol! De repente salió el sol. Juancito se sacó el suéter y lo apoyó en el banco. Luego, su madre se acercó y le entregó un chocolate. Se lo comió de un bocado. Como siempre, al terminarlo, arrojó el envoltorio en el basurero. Había sido una intensa jornada. Se sentía muy cansado. Ya en la falda de su madre, mientras se limpiaba los restos de golosina de la boca, se quedó dormido.

**Neutral text:** La noche recién comenzaba. Alberto estaba eufórico. ¡Gracias a Dios por los fines de semana! A unas pocas cuadras, la discoteca. Sus amigos lo aguardaban allí y juntos compartirían un buen momento. Al cruzar la calle, Alberto leyó el nombre de la discoteca en un cartel: “Ni jefe ni reloj”. Siempre lo ponía de buen humor. Una vez adentro, lo encandilaron las luces. Sintió calor y se encontró muy transpirado. Al lado del bar, un grupo de mujeres se entretenía con las ocurrencias de su amigo, Mario. Las muchachas se reían sin pausa junto a ese joven que bromeaba e inventaba personajes. Luego, entre toda la gente, Alberto reconoció a su novia, Elsa. Ella lo esperaba en una silla. Desde atrás, Alberto le preguntó si le gustaba la música. “¡Por supuesto!”, respondió Elsa. Aunque ella tenía sueño, Alberto le insistió para que lo acompañara a la pista. ¡Era hora de disfrutar la música! Como siempre, al decidirse, Elsa se olvidó la cartera en la silla. Alberto escuchó su canción favorita y se entusiasmó mucho. Elsa, fiel compañera, lo ayudó a recordar la letra. ¡Qué buen equipo! De regreso en su casa, mientras sentía el sudor y el cansancio en el cuerpo, se quedó dormido.

**S3. Texts used in the L2 task**

**Action text:** Donald looked into his bag for his money, but it was not there. Had someone taken it from him? He moved back and forth thinking what to do. He ran to his friend Tommy’s house to take the money he had left there. Tommy was working when Donald rang the bell. He gave him the money and added some more coins. Donald counted the lot in great exultation. Soon after, he went to the newspaper office. He put his money and his coins on the table and asked the receptionist to put an ad in the newspaper the next day. “I have lost my moneybag. If you find it, please call me!”, the ad said. When Donald got up the next day, a man came to his house. He had found the moneybag! Donald thanked the kind man and jumped with exultation. To show his appreciation, he took the man to a bar and they had breakfast together. What a great time it was! Donald had found his moneybag!

**Neutral text:** Poppy lived in a very nice village. The inhabitants there were really nice and supported each other. He was extremely happy and had many friends. However, the inhabitants were very frightened by chocolate. “If you eat it, you’ll be very sick”, a girl said. He thought about this in great curiosity. Was that really true? To find out, he started a journey towards a nearby village, and asked the people there. They found his doubts quite funny and offered him to try some different flavours. How much he loved them! It seemed now difficult to believe the things his friends had told him. During his days there, he ate a lot of chocolate but he never felt sick. He knew the truth now! His friends had believed in a lie when that stranger had come to town. When he went back to his village, he brought a lot of chocolate. He was very proud of himself now. He spent a lot of days teaching people what to believe in.

**S4. Topographical distribution of the data-driven and hypothesis-driven ROIs**


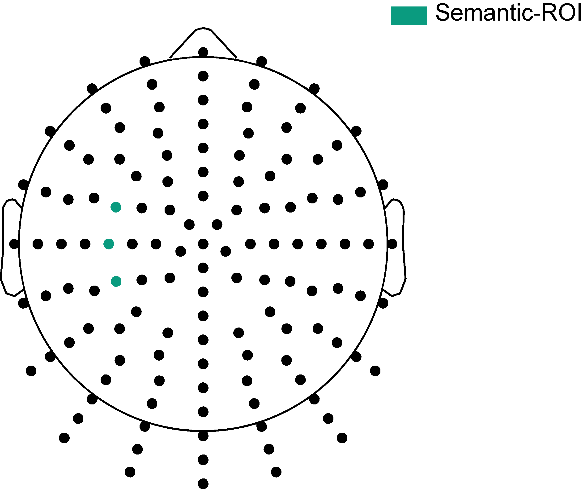

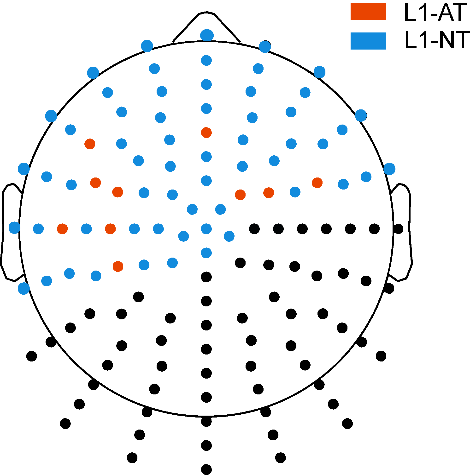

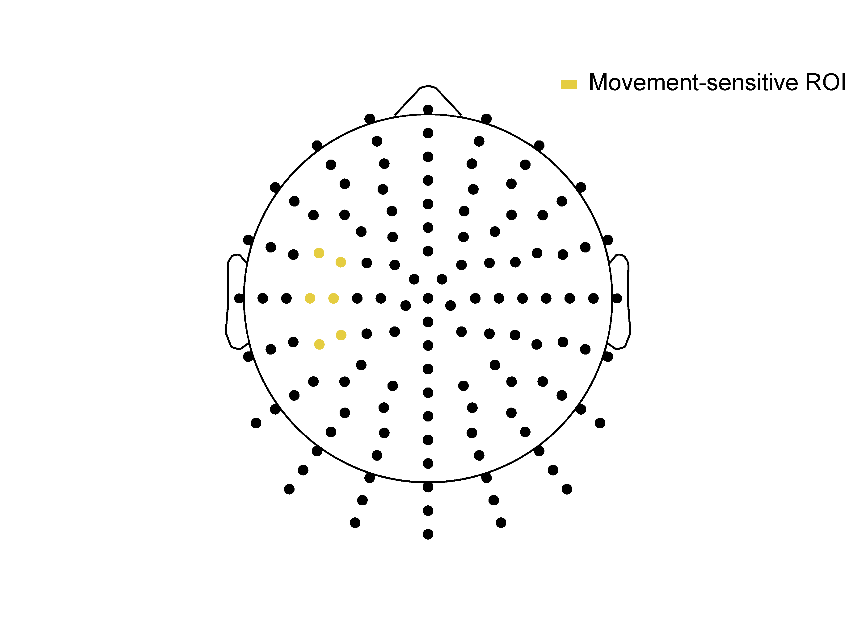


**L1-AT ROI**

**L1-NT ROI**

**Movement-sensitive ROI**

**Embodied ROI**

**A**

**B**

**C**

**Figure S1**. Topographical distribution of the data-driven and hypothesis-driven ROIs. **A.** Data-driven ROIs. Red electrodes integrate the data-driven ROI corresponding to increased functional connectivity for the AT relative to the NT in L1. Blue electrodes integrate the data-driven ROI corresponding to increased functional connectivity for the NT relative to the AT in L1. **B.** Embodied ROI. Green electrodes indicate the embodied ROI, based on results from (Vukovic and Shtyrov, 2014). **C.** Movement-sensitive ROI. Yellow electrodes indicate the movement-sensitive ROI, based on results from Yoris et al. (2017) (Yoris et al., 2017). AT: action text; NT: neutral text; L1: first language; L2: second language; ROI: region of interest.

**S5. Control correlations between AT hyper-connectivity in L2 and L2 proficiency**

L2 proficiency reveled no significant interactions either with hyper-connectivity of the AT-based ROI (*r =* .11, *p =* .56) or the NT-based ROI (*r =* .12, *p =* .53) during L1 (AT vs. NT) processing. The same occurred with the two hypothesis-driven ROIs: L2 proficiency did not significantly correlate with connectivity of the embodied ROI (*r =* -.12, *p =* .55) or the movement-sensitive ROI (*r =* -.13, *p =* .52) during L1 (AT vs. NT) processing.

**S6. Control correlations between AT hyper-connectivity in L2 and age of L2 learning**

Age of L2 learning did not significantly correlate with hyper-connectivity of the AT-based ROI (*r =* -.27, *p =* .186) or the NT-based ROI (*r =* -.21, *p =* .30) during L1 (AT vs. NT) processing. Likewise, there was no significant association between age of L2 learning and connectivity of the embodied ROI (*r =* -.04, *p =* .83) or the movement-sensitive motor ROI (*r =* -.004, *p =* .85) during L1 (AT vs. NT) processing.

Also, as noted in the main manuscript, we reran the correlation between age of L2 learning and connectivity of the data-driven AT ROI after removing a subject whose age of L2 learning (namely, 18) was found to be an outlier. This correlation remained negative and significant (*r =* -.49, *p =* .016, **Figure S2**). Also, all other correlations remained non-significant. Specifically, age of L2 learning did not significantly correlate with either hyper-connectivity of the AT-based ROI (*r =* -.05, *p =* .80) nor with hyper-connectivity of the NT-based ROI (*r =* -.12, *p =* .56) during L1 (AT vs. NT) processing. The same was true of the hypothesis-driven ROIs. Age of L2 learning did not significantly correlate with connectivity of the embodied ROI (*r =* .05, *p =* .81) nor with connectivity of the movement-sensitive motor ROI (*r =* .06, *p =* .77) during L1 (AT vs. NT) processing.


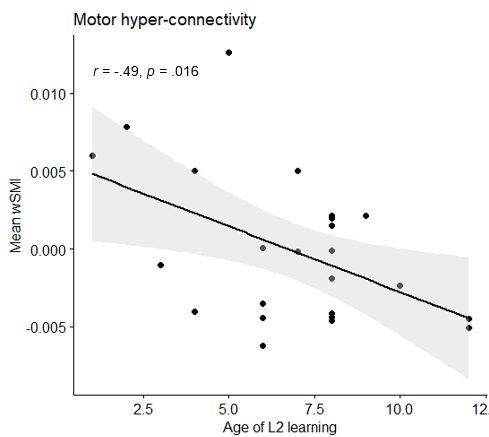


**Figure S2.** Pearson’s correlation between age of L2 learning and L2-AT connectivity based on a data-driven action-grounding ROI after removing an age-of-L2-learning value confirmed to be an outlier at 2 *SD*s from the sample’s mean. The negative correlation remained significant, confirming that the lower the age of L2 learning, the greater the connectivity of the AT ROI during L2 processing.

**Supplementary references**

Vukovic, N., Shtyrov, Y., 2014. Cortical motor systems are involved in second-language comprehension: Evidence from rapid mu-rhythm desynchronisation. *NeuroImage* 102, 695-703.

Yoris, A., García, A.M., Traiber, L., Santamaría-García, H., Martorell, M., Alifano, F., Kichic, R., Moser, J.S., Cetkovich, M., Manes, F., 2017. The inner world of overactive monitoring: neural markers of interoception in obsessive-compulsive disorder. *Psychological* *Medicine* 47, 1957-1970.
